# Osteoporosis 2014

**DOI:** 10.1155/2015/567593

**Published:** 2015-07-28

**Authors:** Ling-Qing Yuan, Hong W. Ouyang, Thomas Levin Andersen, Yebin Jiang, Francesco Pantano, Peng-Fei Shan

**Affiliations:** ^1^Institute of Metabolism and Endocrinology, The Second Xiangya Hospital, Central South University, Changsha 410011, China; ^2^Center for Stem Cell and Tissue Engineering, School of Medicine, Zhejiang University, Hangzhou 310058, China; ^3^Department of Clinical Cell Biology, Vejle Hospital-Lillebaelt Hospital, Institute of Regional Health Science, University of Southern Denmark, 7100 Vejle, Denmark; ^4^University of Michigan Hospital, Ann Arbor, MI 48109, USA; ^5^Medical Oncology Department, Campus Bio-Medico University, 00128 Rome, Italy; ^6^Department of Endocrinology and Metabolism, Second Affiliated Hospital, Zhejiang University College of Medicine, Hangzhou 310009, China

After successfully launching the special issue of “Osteoporosis” in 2013, we are pleased to publish this updated special novel issue. The current issue focuses on the various aspects of advances in osteoporosis, including original articles with both clinical and basic research, as well as reviews. Collectively, the current issue reflects the enormous effort done worldwide to improve the understanding, identification, and treatment of osteoporosis.

*Clinical Studies*. Sarcopenia and osteoporosis are highly prevalent among elderly patients with frailty, which increase the risk of fracture, disability, or even death. Y. Wang et al. performed a cross-sectional analysis in 316 participants aged 65 years and older from Changsha, China, investigating the prevalence of sarcoosteoporosis. Their results show that the prevalence of sarcoosteoporosis is more likely to increase with age and even more so in the elderly with higher levels of comorbidities and with frailty/prefrailty, especially in women. Compared with DXA, quantitative computed tomography (QCT) could examine the true volumetric BMD in three dimensions at any skeletal site. Y. Ma et al. determined lumbar spine and hip volumetric bone mineral density (vBMD) in 826 Chinese adults using QCT. Their results demonstrate that, after achieving peak bone mass (30 to 39 years old in females and 20 to 29 years old in males), the vBMD is decreased with aging. Moreover, their result showed that there is a positive correlation between QCT vBMD and DXA projectional areal BMD (aBMD). Regarding detecting osteoporosis, QCT spine vBMD is more sensitive than CTXA Hip aBMD. J.-H. Huang et al. determined the effect of serum Mg on bone mineral metabolism in CKD patients with or without diabetes. Their study shows that lower serum Mg level results in deficiency in PTH action and exacerbation of osteoporosis in CKD patients, especially those with diabetes. L. Song et al. review the potential mechanisms involved in osteoporosis after organ transplant and demonstrate that combination of vitamin D with bisphosphonates and appropriate dose of glucocorticoids is the most effective protocol to increase BMD of patients with organ transplant, while signaling pathway regulator or BMSC implantation could be a novel direction for the treatment of osteoporosis in patients with organ transplantation. K. Sanders et al. investigated whether vitamin D could decrease fall and fractures. Their study enrolled 2096 females at high risk of fall and/or fracture. The participants completed a prospective 12-month daily fall calendar, which was compared with a 12-month falls recall questionnaire. The conclusion of their study is that “intensive ascertainment of falls is not feasible, 12-month falls recall questions with fewer responses may be an acceptable alternative.” P. Di Carlo et al. reviewed the prevalence of vitamin D deficiency in HIV/HCV coinfected patients. They found lower serum vitamin D in HIV/HCV coinfected patients, which might be associated with progression of live diseases.

*Basic Studies*. Alendronate is a commonly used medication to prevent aseptic loosening with arthroplasty. However, as an oral medication, it has low bioavailability with a long time of administration. D. Song et al. used alendronate in bone cement powder to investigate whether the content of alendronate regulated shear strength of bone-bone cement and metal-bone cement interfaces. Their results reveal that mixed alendronate and bone cement powder could reduce the shear strength at the bone-bone cement interface but not at metal-bone cement interface. K.-J. Kim et al. investigated the effect of Alisol A 24-acetate, a biologically active compound from a traditional Korean herb medicine* Alisma canaliculatum,* on the osteoclastogenesis. Their results demonstrate that Alisol A 24-acetate could decrease the osteoclastogenesis through downregulating NFATc1 and inhibit the expression of DC-STAMP and cathepsin K. They suggested the Alisol A 24-acetate might be a potential scaffold in development of new antiosteoporosis agents. S. Hu et al. used nanoindentation assessment and atomic force microscopy to evaluate the material and structural characteristics of bone in estrogen deficient rat. Their results reveal that estrogen deprivation results in deterioration of structural characteristics but not the nanomechanical properties of the trabecular bone. J.-M. Hou et al.'s study found that lactoferrin promoted osteoblast proliferation while it inhibited apoptosis through IGF-1R.

There are ongoing progresses in osteoporosis research, and the present special issue covers only some areas of new developments.

